# Behçet's syndrome treated with infliximab, which caused a palmoplantar pustulosis, subsequently maintained on low-dose etanercept

**DOI:** 10.3402/ljm.v7i0.19139

**Published:** 2012-10-11

**Authors:** Amani Tresh, Victoria H. Smith, Richard A.G. Parslew

**Affiliations:** 1 Dermatology Department, Bir Usta Milad Hospital, Tripoli, Libya; 2 Dermatology Department, Royal Liverpool and Broadgreen University Hospitals, Liverpool, United Kingdom

Behçet's syndrome is a chronic, relapsing, multisystemic, inflammatory disorder. The diagnosis of Behçet's syndrome is based on clinical criteria and no pathognomonic laboratory findings exist. A diagnosis is made by the presence of recurrent oral ulceration, the hallmark of this disease, plus any two of the following: recurrent genital ulcerations, ocular lesions (anterior or posterior uveitis, cells in vitreous or slit lamp examination, or retinal vasculitis), typical skin lesions, and a positive pathergy (skin hyperreactivity) test (1). Inflammation in Behçet's disease is thought to be mediated by cytokines derived from T-helper type 1 lymphocytes, including tumor necrosis factor (TNF) (2). In the absence of the multisystem disease, aphthae may be treated topically with topical steroid, topical tacrolimus, and/or with topical lidocaine (3). Mucocutaneous manifestations may respond to colchicine or dapsone alone or in combination (3). Oral corticosteroid may not control severe disease where upon methotrexate (4), azathioprine (5) cyclosporine (6), tacrolimus (7), chlorambucil (4), or cyclophosphamide (4) need to be considered. Thalidomide can be used with significant clinical benefit but is frequently complicated by peripheral neuropathy (8). Interferon α is also an effective alternative treatment for the mucocutaneous manifestations (9). Recently, the anti-TNF-α agents have successfully been used in treating resistant Behçet's syndrome. Etanercept, a human TNF receptor fusion protein, has shown to be beneficial for the resistant cases at a dose of 25 mg twice weekly (2). Infliximab, a chimeric monoclonal antibody against TNF-α, has also been used effectively in therapy-resistant Behçet's disease, including cases resistant to etanercept (10).

## Case report

We report a 55-year-old woman with Behçet's syndrome who is on a low-dose etanercept, following paradoxical pustular psoriasis associated with infliximab, which cleared with superficial radiotherapy. Her palmoplanter pustular psoriasis, which was also induced at a higher dose etanercept interestingly disappeared.

She was diagnosed in 2001 with Behçet's based on recurrent oral and genital ulceration, large joint arthritis, cutaneous vasculitis, and positive pathergy phenomenon. Investigations revealed no evidence of autoimmune diseases or inflammatory bowel disease. She had also an episode of aseptic meningitis. Her condition has been non-compliant to multiple treatments: dapsone, colchicine, pentoxifylline, methotrexate, and intravenous immunoglobulin. Other systemic treatments used have benefits. However, these treatments were stopped due to many side effects, including pulsed cyclophosphamide, cyclosporine, mycophenolate mofetil, and clofazimine. She responded well to systemic steroids but developed severe osteoporosis. She also tolerated and responded very well to thalidomide (100 mg daily) for more than a year. However, with the detection of a peripheral neuropathy on nerve conduction studies, this also had to be discontinued.

**Fig. 1:**
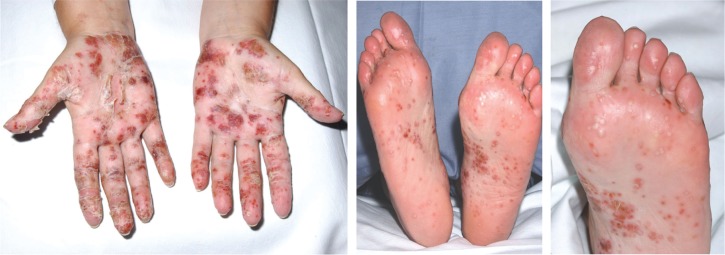
The acute eruption of painful pustules on the palms and soles.

**Fig. 2:**
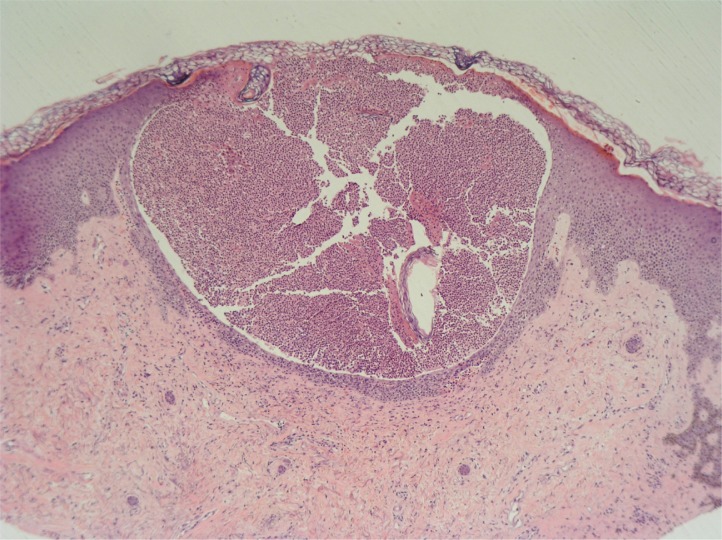
Histological examination showing an intradermal pustule and a mild perivascular lymphocytic infiltrate, with no true vasculitis or psorasiform features (H&E stain).

In June 2006, she commenced infliximab infusion (5 mg/kg), and 6 weeks after her third infusion she developed a painful pustular eruption on the palms and soles (Fig. 1). Histology showed an intradermal pustule, a mild perivascular lymphocytic infiltrate but no true vasculitis, and there was no psorasiform feature (Fig. 2). Since there were no other clinical or histological features of a flare of Behçet's, no family history of psoriasis, and no features of a bacterial infection, ruling out pustular bacterid, a diagnosis of infliximab-induced palmoplantar pustulosis (PPP) was made. Her PPP responded to hand and foot superficial radiotherapy (5 grays in 5 fractions). This is one of the effective ways of treating refractory PPP (11), having failed very potent topical steroid and topical PUVA alone and combined with acitretin, which are the main therapeutic modalities for PPP. Consequently, in October 2006, she started on twice-weekly subcutaneous injections of 25 mg etanercept. Following this, she had no further relapse of her Behçet's syndrome. However, her PPP recurred, and this has gradually improved following patient self-titration of the etanercept dose. Currently she is asympothomatic on etanercept (10 mg) every 10 days.

## Discussion

Our case has supported the previous literature, which has shown adverse skin manifestations secondary to TNF-α inhibitors. Various adverse reactions involving the skin, such as a rash, pruritus, urticaria, dry skin, fungal dermatitis, onychomycosis, eczema, and bullous eruptions have frequently been described in patients with rheumatoid arthritis or Crohn's disease treated with infliximab (12). One hundred and twenty patients have been reported to develop pustular lesions during treatment with TNF-α inhibitors (13). Psoriasis (except palmo plantar pustular type) was the most common adverse effect during anti-TNF-α treatment (*n*=73), followed by palmoplantar pustular psoriasis (*n*=37) and psoriasis of the nail (*n*=6), sometimes combined in the same patient. PPP and psoriasiform exanthema were diagnosed in 10 patients. It has been suggested that PPP in this context should be regarded as an adverse outcome of anti-TNF-α treatment (14). It has already been hypothesized that there is polymorphism of the TNF-α gene in patients with Behçet's syndrome (15). These polymorphisms may account for our patient's response to low-dose etanercept, but at higher doses her PPP is induced. However, our experience is limited due to lack of relevant literature.

*Amani Tresh* Dermatology Department Bir Usta Milad Hospital Tripoli, Libya Email: treshamani@yahoo.co.uk*Victoria H. Smith and Richard A.G. Parslew* Dermatology Department Royal Liverpool and Broadgreen University Hospitals Liverpool, United Kingdom

## References

[1] International Study Group for Behçet's disease (1990). Criteria for diagnosis of Behçet's disease. Lancet.

[2] Sfikakis PP (2002). Behçet's disease: a new target for anti-tumour necrosis factor treatment. Ann Rheum Dis.

[3] Barham K, Jorizzo J, Grattan B, Cox N (2004). Rook text book of dermatology.

[4] Kaklamani VG, Kaklamanis PG (2001). Treatment of Behçet's disease: an update. Semin Arthritis Rheum.

[5] Yazici H, Pazarli H, Barnes CG, Tüzün Y, Ozyazgan Y, Silman A (1990). A controlled trial of azathioprine in Behçet's syndrome. N Engl J Med.

[6] Masuda K, Nakajima A, Urayama A, Nakae K, Kogure M, Inaba G (1989). Double-masked trial of cyclosporin versus colchicine and long-term open study of cyclosporin in Behçet's disease. Lancet.

[7] Ishioka M, Ohno S, Nakamura S, Isobe K, Watanabe N, Ishigatsubo Y (1994). FK506 treatment of non infectious uveitis. Am J Ophthalmol.

[8] Hamaryudan V, Mat C, Saip S, Ozyazgan Y, Siva A, Yurdakul S (1998). Thalidomide in the treatment of the mucocutaneous lesions of the Behçet's syndrome. A randomized, double blind, placebo controlled trial. Ann Intern Med.

[9] Alpsoy E, Durusoy C, Yilmaz E, Ozgurel Y, Ermis O, Yazar S (2002). Interferon alfa-2a in the treatment of Behçet's disease: a randomized placebo-controlled and double-blind study. Arch Dermatol.

[10] Estrach C, Mpofu S, Moots RJ (2002). Behçet's syndrome: response to infliximab after failure of etanercept. Rheumatology.

[11] Sumila M, Notter M, Itin P, Bodis S, Gruber G (2008). Long-term results of radiotherapy in patients with chronic palmo-plantar eczema or psoriasis. Strahlenther Onkol.

[12] European, Agency, for, et al http://www.emea.eu.int/pdfs/human/press/pus/444500en.pdf.

[13] Wollina U, Hansel G, Koch A, Schönlebe J, Köstler E, Haroske G (2008). Tumor necrosis factor-alpha inhibitor-induced psoriasis or psoriasiform exanthemata: first 120 cases from the literature including a series of six new patients. Am J Clin Dermatol.

[14] Sfikakis PP, Iliopoulos A, Elezoglou A, Kittas C, Stratigos A (2005). Psoriasis induced by anti-tumour necrosis factor therapy: a paradoxical adverse reaction. Arthritis Rheum.

[15] Dobson CM, Parslew RAG (2003). Exacerbation of psoriasis by thalidomide in Behçet's syndrome. Br J Dermatol.

